# 1-(Phthalimidometh­yl)pyridinium *p*-toluene­sulfonate

**DOI:** 10.1107/S1600536808038816

**Published:** 2008-12-10

**Authors:** Mark Daniel Bartholomä, Wayne Ouellette, Jon Zubieta

**Affiliations:** a Department of Chemistry, Syracuse University, Syracuse, New York 13244, USA

## Abstract

In the crystal of the title compound, C_14_H_11_N_2_O_2_^+^·C_7_H_7_O_3_S^−^, the cation and anion inter­act by way of an aromatic π–π inter­action [centroid–centroid separation = 3.5783 (2) Å] and a T-stacking (C—H⋯π) inter­action between cations. The dihedral angle between the aromatic rings in the cation is 61.73 (8)°. The ionic units are aligned in a zigzag fashion in the *b*-axis direction.

## Related literature

For medicinal background, see: Al-Madhoun *et al.* (2002 ▶); Arner & Eriksson (1995 ▶); Bello (1974 ▶); Celen *et al.* (2007 ▶); Eriksson *et al.* (2002 ▶); Wei *et al.* (2005 ▶); Welin *et al.* (2004 ▶).
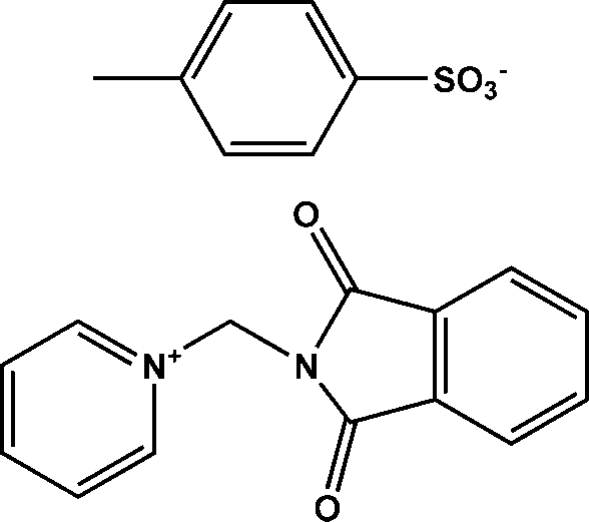


## Experimental

### 

#### Crystal data

C_14_H_11_N_2_O_2_^+^·C_7_H_7_O_3_S^−^
*M* *_r_* = 410.43
Monoclinic, 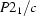

*a* = 7.6944 (5) Å
*b* = 33.626 (2) Å
*c* = 7.9426 (5) Å
β = 116.416 (1)°
*V* = 1840.5 (2) Å^3^
*Z* = 4
Mo *K*α radiation
μ = 0.21 mm^−1^
*T* = 90 (2) K
0.30 × 0.25 × 0.20 mm

#### Data collection

Bruker APEX CCD diffractometer
Absorption correction: multi-scan (*SADABS*; Sheldrick, 2008 ▶) *T*_min_ = 0.939, *T*_max_ = 0.958
19080 measured reflections
4482 independent reflections
3753 reflections with *I* > 2σ(*I*)
*R*_int_ = 0.049

#### Refinement

*R*[*F* ^2^ > 2σ(*F* ^2^)] = 0.054
*wR*(*F* ^2^) = 0.124
*S* = 1.12
4482 reflections
263 parameters
H-atom parameters constrained
Δρ_max_ = 0.61 e Å^−3^
Δρ_min_ = −0.38 e Å^−3^

### 

Data collection: *SMART* (Bruker, 2002 ▶); cell refinement: *SAINT* (Bruker, 2002 ▶); data reduction: *SAINT*; program(s) used to solve structure: *SHELXS97* (Sheldrick, 2008 ▶); program(s) used to refine structure: *SHELXL97* (Sheldrick, 2008 ▶); molecular graphics: *DIAMOND* (Brandenburg & Putz (1999 ▶); software used to prepare material for publication: *SHELXTL* (Sheldrick, 2008 ▶).

## Supplementary Material

Crystal structure: contains datablocks I, global. DOI: 10.1107/S1600536808038816/hb2840sup1.cif

Structure factors: contains datablocks I. DOI: 10.1107/S1600536808038816/hb2840Isup2.hkl

Additional supplementary materials:  crystallographic information; 3D view; checkCIF report

## Figures and Tables

**Table 1:** Hydrogen-bond geometry (Å, °)

*D*—H⋯*A*	*D*—H	H⋯*A*	*D*⋯*A*	*D*—H⋯*A*
C5—H5⋯*Cg*1^i^	0.95	2.70	3.531 (2)	147

Symmetry code: (i) 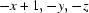. *Cg*1 is the centroid of the N2/C10–C14 ring.

**Fig. 1.**
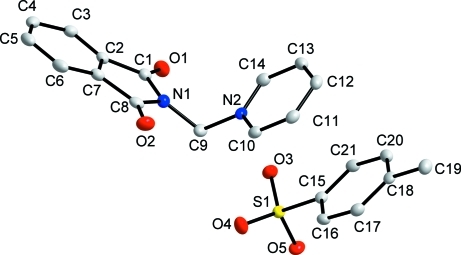
Perspective view of (I), with displacement ellipsoids drawn at 50% probability level (H atoms omitted for clarity).

**Fig. 2.**
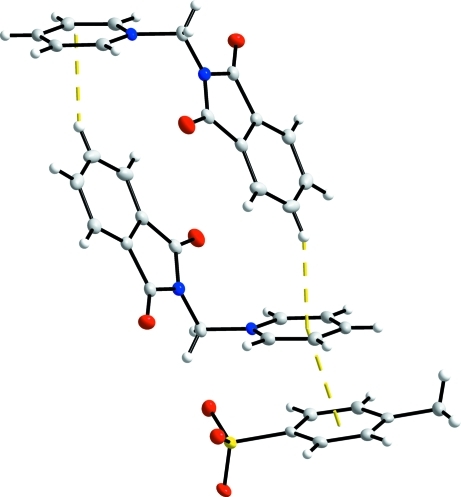
Aromatic interactions observed within the crystal lattice of (I).

**Fig. 3.**
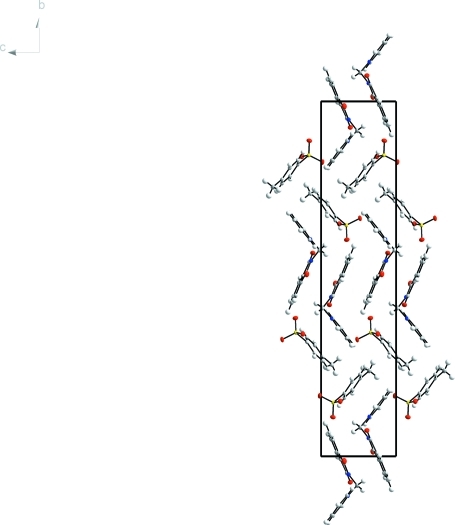
The crystal packing of (I) viewed parallel to the bc plane.

## supplementary crystallographic information

### Comment

Radiolabeled nucleosides and nucleoside analogs may be good candidates for imaging and therapeutic applications because of their metabolic entrapment in rapidly dividing cells like tumor cells. These radiolabeled nucleoside derivatives may act as substrates for the human cytosolic thymidine kinase (hTK-1), an enzyme of the salvage pathway which catalyzes the phosphorylation of nucleosides to their corresponding 5'-monophosphates (Welin *et al.*, 2004). The phosphorylation would mainly occur in proliferating tumor cells since hTK-1 shows a dramatically increased activity in tumor cells compared to quiescent cells (Bello, 1974). The phosphorylated nucleosides would be entrapped inside the proliferating cells because of their negatively charged phosphate moiety retarding the cellular efflux (Arner *et al.*, 1995). Thus, a radiolabeled nucleoside analog could be used as probe for tumor cell proliferation since the entrapment results in an accumulation in tissue with elevated hTK-1 activity. The main problem for the development of a suitable nucleoside analog lies in the narrow substrate specifity of hTK-1 (Eriksson *et al.*, 2002). The natural substrates for hTK-1 are thymidine and uridine. Major modifications of the corresponding nucleoside, however, may lead to a highly decreased activity. The literature on the interaction of thymidine derivatives with hTK-1 is not totally unambiguous about the effects of various substitutions. For example, N-3 derivatized thymidine analogs have been reported to be inactive (Celen *et al.*, 2007). On the other hand, N-3 modified carboranylalkyl thymidine analogs show acceptable conversion rates (Al-Madhoun *et al.*, 2002). Therefore, we made a set of several thymidine and uridine analogs modified at different positions of the ribose and the base moiety to get further insight on the effects of various derivatizations. To expand our SAAC concept (single amino acid chelate) for radioimaging and radiotherapeutic purposes on nucleosides, the title phthalimidomethylpyridinium *p*-toluenesulfonate salt, (I), was prepared as part of a series of tosylalkylphthalimide derivatives recently synthesized in our group. This series is used for the attachment of a SAAC chelate at the N-3 and C-5 position of the base moiety of thymidine (Bartholomä *et al.* unpublished results). The SAAC chelate allows hereby the radiolabelling of thymidine and uridine derivatives by the coordination of the [*M*(CO)_3_]^+^ core (*M* = ^186/188^Re, ^99^*^m^*Tc) (Wei *et al.*, 2005). The ideal decay properties, low cost and convenient availability of ^99^*^m^*Tc from generator columns make the corresponding nucleoside complexes interesting candidates for imaging purposes while their corresponding rhenium complexes could be used as therapeutic counterparts.

Due to the tetrahedral arrangement of the connecting methyl group, the phthalimidomethylpyridinium cation in (I) is not planar. The tosylate anion sits on top of one end of the pyridinium residue showing aromatic π-π interaction. The centroid distance between those two aromatic rings is 3.5783 (2) Å. The other end of the pyridinium moiety shows some interaction with the phthalimide part of neighbored phthalimidomethyl-pyridinium cation. Thus, a T-stacking between the pyridinium residue and the benzyl ring of the phthalimide residue occurs (Table 1). The distances between the interacting C—H of the phthalimide and the centroid of the pyridinium residue are C2–Centroid = 3.5313 (2) Å and H2–Centroid = 2.70Å, respectively. The corresponding angle C2–H2···Centroid is 147°. The phthalimide moiety itself has a planar geometry. All bond length and angles fall in expected ranges. In the crystal, the ionic units are aligned in a zigzag arrangement in direction of the *b* axis.

### Experimental

2.00 g (11.29 mmol) *N*-(Hydroxymethyl)phthalimide were dissolved in 20 ml anhydrous pyridine under an inert atmosphere followed by a dropwise addition of 3.23 g (16.93 mmol, 1.5 equiv.) *p*-Toluenesulfonyl chloride in 20 ml anhydrous pyridine. After complete addition of the tosylchloride, the reaction mixture was stirred for additional 16 h. About 2 h after the addition was completed, a white precipitate started to form. This white solid was filtered off, washed three times with 100 ml chloroform, and finally dried for several days at h.v.. The product was obtained in good yields as a colourless amorphous powder (3.80 g, quantitative); colourless blocks of (I) suitable for X-ray diffraction were collected directly from the reaction mixture. ^1^H NMR (d_6_-DMSO): *δ* = 2.28 (s, 3 H), 6.41 (s, 2 H), 7.10 (d, J = 7.98 Hz, 2 H), 7.47 (d, J = 7.95 Hz, 2 H), 7.90–7.99 (m, 4 H), 8.20 (t, J = 7.05 Hz, 2 H), 8.67 (t, J = 7.68 Hz, 1 H), 9.09 (d, J = 5.80 Hz, 2 H). p.p.m.. IR: *ν* = 3398 (br), 3124, 3087, 3037, 2980, 2935, 1781, 1729, 1627, 1485, 1404, 1361, 1331, 1300, 1268, 1206, 1168, 1116, 1089, 1067, 1031, 1008, 951, 826, 801, 777, 728, 680, 632, 587, 565, 529 cm^-1^.

### Refinement

The H atoms were placed in calculated positions and refined as riding.

### Crystal data

**Table:**

C_14_H_11_N_2_O_2_^+^·C_7_H_7_O_3_S^−^	*F*(000) = 856
*M**_r_* = 410.43	*D*_x_ = 1.481 Mg m^−^^3^
Monoclinic, *P*2_1_/*c*	Mo *K*α radiation, λ = 0.71073 Å
Hall symbol: -P 2ybc	Cell parameters from 3297 reflections
*a* = 7.6944 (5) Å	θ = 2.4–26.4°
*b* = 33.626 (2) Å	µ = 0.21 mm^−^^1^
*c* = 7.9426 (5) Å	*T* = 90 K
β = 116.416 (1)°	Block, colourless
*V* = 1840.5 (2) Å^3^	0.30 × 0.25 × 0.20 mm
*Z* = 4	

### Data collection

**Table:**

Bruker APEX CCD diffractometer	4482 independent reflections
Radiation source: fine-focus sealed tube	3753 reflections with *I* > 2σ(*I*)
graphite	*R*_int_ = 0.049
Detector resolution: 512 pixels mm^-1^	θ_max_ = 28.1°, θ_min_ = 2.4°
ω scans	*h* = −10→10
Absorption correction: multi-scan (*SADABS*; Sheldrick, 2008)	*k* = −42→44
*T*_min_ = 0.939, *T*_max_ = 0.958	*l* = −10→10
19080 measured reflections	

### Refinement

**Table:**

Refinement on *F*^2^	Primary atom site location: structure-invariant direct methods
Least-squares matrix: full	Secondary atom site location: difference Fourier map
*R*[*F*^2^ > 2σ(*F*^2^)] = 0.054	Hydrogen site location: inferred from neighbouring sites
*wR*(*F*^2^) = 0.124	H-atom parameters constrained
*S* = 1.12	*w* = 1/[σ^2^(*F*_o_^2^) + (0.0508*P*)^2^ + 1.1529*P*] where *P* = (*F*_o_^2^ + 2*F*_c_^2^)/3
4482 reflections	(Δ/σ)_max_ < 0.001
263 parameters	Δρ_max_ = 0.61 e Å^−^^3^
0 restraints	Δρ_min_ = −0.38 e Å^−^^3^

### Special details

**Table:**

Geometry. All e.s.d.'s (except the e.s.d. in the dihedral angle between two l.s. planes) are estimated using the full covariance matrix. The cell e.s.d.'s are taken into account individually in the estimation of e.s.d.'s in distances, angles and torsion angles; correlations between e.s.d.'s in cell parameters are only used when they are defined by crystal symmetry. An approximate (isotropic) treatment of cell e.s.d.'s is used for estimating e.s.d.'s involving l.s. planes.
Refinement. Refinement of *F*^2^ against ALL reflections. The weighted *R*-factor *wR* and goodness of fit *S* are based on *F*^2^, conventional *R*-factors *R* are based on *F*, with *F* set to zero for negative *F*^2^. The threshold expression of *F*^2^ > σ(*F*^2^) is used only for calculating *R*-factors(gt) *etc*. and is not relevant to the choice of reflections for refinement. *R*-factors based on *F*^2^ are statistically about twice as large as those based on *F*, and *R*- factors based on ALL data will be even larger.

### Fractional atomic coordinates and isotropic or equivalent isotropic displacement parameters (Å^2^)

**Table:**

	*x*	*y*	*z*	*U*_iso_*/*U*_eq_	
S1	0.71561 (7)	0.151380 (15)	0.83629 (7)	0.01495 (13)	
O1	1.0063 (2)	0.07297 (5)	0.3896 (2)	0.0232 (3)	
O2	0.4402 (2)	0.01341 (5)	0.3092 (2)	0.0247 (4)	
O3	0.8408 (2)	0.15516 (5)	0.7439 (2)	0.0209 (3)	
O4	0.6566 (2)	0.11048 (4)	0.8427 (2)	0.0232 (3)	
O5	0.7929 (2)	0.17090 (5)	1.0188 (2)	0.0217 (3)	
N1	0.7117 (2)	0.05127 (5)	0.3668 (2)	0.0173 (4)	
N2	0.5370 (2)	0.11140 (5)	0.3597 (2)	0.0158 (4)	
C1	0.8823 (3)	0.04795 (6)	0.3432 (3)	0.0169 (4)	
C2	0.8696 (3)	0.00828 (6)	0.2554 (3)	0.0159 (4)	
C3	0.9997 (3)	−0.01049 (6)	0.2060 (3)	0.0191 (4)	
H3	1.1166	0.0020	0.2219	0.023*	
C4	0.9513 (3)	−0.04884 (7)	0.1314 (3)	0.0228 (5)	
H4	1.0367	−0.0628	0.0952	0.027*	
C5	0.7805 (3)	−0.06681 (7)	0.1096 (3)	0.0239 (5)	
H5	0.7523	−0.0930	0.0598	0.029*	
C6	0.6488 (3)	−0.04768 (7)	0.1584 (3)	0.0217 (4)	
H6	0.5315	−0.0601	0.1423	0.026*	
C7	0.6972 (3)	−0.00982 (6)	0.2314 (3)	0.0170 (4)	
C8	0.5931 (3)	0.01743 (6)	0.3028 (3)	0.0179 (4)	
C9	0.6823 (3)	0.08173 (6)	0.4785 (3)	0.0204 (4)	
H9A	0.8071	0.0953	0.5547	0.025*	
H9B	0.6379	0.0694	0.5657	0.025*	
C10	0.3571 (3)	0.11045 (6)	0.3473 (3)	0.0177 (4)	
H10	0.3245	0.0910	0.4150	0.021*	
C11	0.2198 (3)	0.13761 (6)	0.2367 (3)	0.0182 (4)	
H11	0.0917	0.1367	0.2257	0.022*	
C12	0.2708 (3)	0.16635 (6)	0.1417 (3)	0.0193 (4)	
H12	0.1781	0.1855	0.0657	0.023*	
C13	0.4584 (3)	0.16703 (6)	0.1581 (3)	0.0192 (4)	
H13	0.4952	0.1867	0.0942	0.023*	
C14	0.5902 (3)	0.13900 (6)	0.2678 (3)	0.0185 (4)	
H14	0.7184	0.1390	0.2790	0.022*	
C15	0.4978 (3)	0.17744 (6)	0.6922 (3)	0.0147 (4)	
C16	0.3230 (3)	0.16559 (6)	0.6887 (3)	0.0170 (4)	
H16	0.3196	0.1430	0.7591	0.020*	
C17	0.1535 (3)	0.18648 (6)	0.5831 (3)	0.0180 (4)	
H17	0.0355	0.1783	0.5832	0.022*	
C18	0.1543 (3)	0.21947 (6)	0.4764 (3)	0.0169 (4)	
C19	−0.0316 (3)	0.24117 (7)	0.3577 (3)	0.0221 (5)	
H19A	−0.0026	0.2652	0.3049	0.033*	
H19B	−0.0957	0.2486	0.4357	0.033*	
H19C	−0.1176	0.2238	0.2553	0.033*	
C20	0.3304 (3)	0.23071 (6)	0.4797 (3)	0.0184 (4)	
H20	0.3336	0.2529	0.4069	0.022*	
C21	0.5012 (3)	0.21025 (6)	0.5872 (3)	0.0167 (4)	
H21	0.6198	0.2186	0.5889	0.020*	

### Atomic displacement parameters (Å^2^)

**Table:**

	*U*^11^	*U*^22^	*U*^33^	*U*^12^	*U*^13^	*U*^23^
S1	0.0132 (2)	0.0163 (2)	0.0136 (2)	−0.00043 (18)	0.00446 (18)	−0.00117 (18)
O1	0.0220 (8)	0.0201 (8)	0.0253 (8)	−0.0060 (6)	0.0086 (7)	−0.0012 (6)
O2	0.0170 (7)	0.0361 (9)	0.0229 (8)	−0.0006 (7)	0.0105 (6)	0.0016 (7)
O3	0.0184 (7)	0.0250 (8)	0.0212 (8)	0.0008 (6)	0.0106 (6)	−0.0010 (6)
O4	0.0203 (8)	0.0182 (8)	0.0288 (8)	−0.0001 (6)	0.0087 (7)	0.0030 (6)
O5	0.0183 (7)	0.0290 (8)	0.0130 (7)	0.0020 (6)	0.0027 (6)	−0.0037 (6)
N1	0.0168 (8)	0.0177 (9)	0.0160 (8)	0.0022 (7)	0.0060 (7)	0.0003 (7)
N2	0.0154 (8)	0.0182 (8)	0.0110 (8)	0.0043 (7)	0.0035 (7)	−0.0007 (6)
C1	0.0165 (10)	0.0187 (10)	0.0135 (9)	0.0029 (8)	0.0049 (8)	0.0032 (7)
C2	0.0181 (10)	0.0150 (9)	0.0120 (9)	0.0015 (8)	0.0044 (8)	0.0032 (7)
C3	0.0207 (10)	0.0221 (11)	0.0144 (10)	0.0022 (8)	0.0077 (8)	0.0034 (8)
C4	0.0315 (12)	0.0237 (11)	0.0137 (10)	0.0088 (9)	0.0105 (9)	0.0037 (8)
C5	0.0371 (13)	0.0169 (10)	0.0133 (10)	−0.0018 (9)	0.0074 (9)	0.0002 (8)
C6	0.0262 (11)	0.0208 (11)	0.0149 (10)	−0.0056 (9)	0.0063 (9)	0.0012 (8)
C7	0.0158 (9)	0.0192 (10)	0.0132 (9)	0.0000 (8)	0.0041 (8)	0.0036 (7)
C8	0.0167 (10)	0.0220 (10)	0.0119 (9)	0.0004 (8)	0.0036 (8)	0.0047 (8)
C9	0.0212 (10)	0.0241 (11)	0.0129 (9)	0.0076 (8)	0.0047 (8)	0.0020 (8)
C10	0.0193 (10)	0.0194 (10)	0.0144 (9)	−0.0002 (8)	0.0076 (8)	−0.0026 (8)
C11	0.0148 (9)	0.0223 (10)	0.0149 (10)	0.0009 (8)	0.0044 (8)	−0.0050 (8)
C12	0.0198 (10)	0.0186 (10)	0.0142 (10)	0.0044 (8)	0.0026 (8)	−0.0026 (8)
C13	0.0247 (11)	0.0171 (10)	0.0152 (10)	0.0006 (8)	0.0083 (8)	0.0006 (8)
C14	0.0177 (10)	0.0207 (10)	0.0163 (10)	−0.0010 (8)	0.0068 (8)	−0.0031 (8)
C15	0.0139 (9)	0.0166 (9)	0.0102 (9)	0.0000 (7)	0.0022 (7)	−0.0026 (7)
C16	0.0199 (10)	0.0185 (10)	0.0123 (9)	−0.0001 (8)	0.0068 (8)	0.0001 (7)
C17	0.0163 (9)	0.0213 (10)	0.0158 (10)	0.0002 (8)	0.0067 (8)	−0.0027 (8)
C18	0.0205 (10)	0.0159 (9)	0.0107 (9)	0.0013 (8)	0.0038 (8)	−0.0042 (7)
C19	0.0207 (11)	0.0228 (11)	0.0184 (10)	0.0049 (8)	0.0047 (9)	−0.0019 (8)
C20	0.0233 (10)	0.0155 (10)	0.0139 (9)	−0.0002 (8)	0.0061 (8)	−0.0010 (7)
C21	0.0162 (9)	0.0188 (10)	0.0134 (9)	−0.0037 (8)	0.0050 (8)	−0.0035 (7)

### Geometric parameters (Å, °)

**Table:**

S1—O3	1.4531 (15)	C9—H9B	0.9900
S1—O5	1.4556 (15)	C10—C11	1.377 (3)
S1—O4	1.4562 (16)	C10—H10	0.9500
S1—C15	1.783 (2)	C11—C12	1.386 (3)
O1—C1	1.201 (3)	C11—H11	0.9500
O2—C8	1.208 (2)	C12—C13	1.391 (3)
N1—C8	1.405 (3)	C12—H12	0.9500
N1—C1	1.410 (3)	C13—C14	1.375 (3)
N1—C9	1.437 (3)	C13—H13	0.9500
N2—C10	1.343 (3)	C14—H14	0.9500
N2—C14	1.352 (3)	C15—C21	1.390 (3)
N2—C9	1.482 (3)	C15—C16	1.391 (3)
C1—C2	1.488 (3)	C16—C17	1.387 (3)
C2—C3	1.380 (3)	C16—H16	0.9500
C2—C7	1.393 (3)	C17—C18	1.397 (3)
C3—C4	1.399 (3)	C17—H17	0.9500
C3—H3	0.9500	C18—C20	1.396 (3)
C4—C5	1.386 (3)	C18—C19	1.506 (3)
C4—H4	0.9500	C19—H19A	0.9800
C5—C6	1.393 (3)	C19—H19B	0.9800
C5—H5	0.9500	C19—H19C	0.9800
C6—C7	1.380 (3)	C20—C21	1.390 (3)
C6—H6	0.9500	C20—H20	0.9500
C7—C8	1.486 (3)	C21—H21	0.9500
C9—H9A	0.9900		
			
O3—S1—O5	113.25 (9)	H9A—C9—H9B	108.0
O3—S1—O4	112.78 (9)	N2—C10—C11	120.30 (19)
O5—S1—O4	112.75 (10)	N2—C10—H10	119.8
O3—S1—C15	106.13 (9)	C11—C10—H10	119.8
O5—S1—C15	105.58 (9)	C10—C11—C12	119.12 (19)
O4—S1—C15	105.53 (9)	C10—C11—H11	120.4
C8—N1—C1	112.38 (17)	C12—C11—H11	120.4
C8—N1—C9	123.06 (18)	C11—C12—C13	119.60 (19)
C1—N1—C9	123.35 (18)	C11—C12—H12	120.2
C10—N2—C14	121.75 (18)	C13—C12—H12	120.2
C10—N2—C9	119.46 (18)	C14—C13—C12	119.4 (2)
C14—N2—C9	118.78 (17)	C14—C13—H13	120.3
O1—C1—N1	124.31 (19)	C12—C13—H13	120.3
O1—C1—C2	130.5 (2)	N2—C14—C13	119.82 (19)
N1—C1—C2	105.22 (17)	N2—C14—H14	120.1
C3—C2—C7	121.9 (2)	C13—C14—H14	120.1
C3—C2—C1	129.71 (19)	C21—C15—C16	119.42 (18)
C7—C2—C1	108.40 (18)	C21—C15—S1	120.78 (15)
C2—C3—C4	116.9 (2)	C16—C15—S1	119.78 (15)
C2—C3—H3	121.6	C17—C16—C15	120.50 (19)
C4—C3—H3	121.6	C17—C16—H16	119.8
C5—C4—C3	120.9 (2)	C15—C16—H16	119.8
C5—C4—H4	119.5	C16—C17—C18	120.81 (19)
C3—C4—H4	119.5	C16—C17—H17	119.6
C4—C5—C6	122.1 (2)	C18—C17—H17	119.6
C4—C5—H5	119.0	C20—C18—C17	118.02 (19)
C6—C5—H5	119.0	C20—C18—C19	121.63 (19)
C7—C6—C5	116.7 (2)	C17—C18—C19	120.33 (19)
C7—C6—H6	121.7	C18—C19—H19A	109.5
C5—C6—H6	121.7	C18—C19—H19B	109.5
C6—C7—C2	121.6 (2)	H19A—C19—H19B	109.5
C6—C7—C8	129.7 (2)	C18—C19—H19C	109.5
C2—C7—C8	108.70 (18)	H19A—C19—H19C	109.5
O2—C8—N1	124.3 (2)	H19B—C19—H19C	109.5
O2—C8—C7	130.4 (2)	C21—C20—C18	121.47 (19)
N1—C8—C7	105.31 (17)	C21—C20—H20	119.3
N1—C9—N2	111.61 (16)	C18—C20—H20	119.3
N1—C9—H9A	109.3	C20—C21—C15	119.78 (19)
N2—C9—H9A	109.3	C20—C21—H21	120.1
N1—C9—H9B	109.3	C15—C21—H21	120.1
N2—C9—H9B	109.3		
			
C8—N1—C1—O1	−179.12 (19)	C1—N1—C9—N2	108.7 (2)
C9—N1—C1—O1	−11.4 (3)	C10—N2—C9—N1	104.0 (2)
C8—N1—C1—C2	0.2 (2)	C14—N2—C9—N1	−76.5 (2)
C9—N1—C1—C2	167.94 (17)	C14—N2—C10—C11	1.0 (3)
O1—C1—C2—C3	0.9 (4)	C9—N2—C10—C11	−179.58 (18)
N1—C1—C2—C3	−178.3 (2)	N2—C10—C11—C12	−1.3 (3)
O1—C1—C2—C7	179.2 (2)	C10—C11—C12—C13	0.6 (3)
N1—C1—C2—C7	−0.1 (2)	C11—C12—C13—C14	0.4 (3)
C7—C2—C3—C4	−0.4 (3)	C10—N2—C14—C13	0.0 (3)
C1—C2—C3—C4	177.63 (19)	C9—N2—C14—C13	−179.44 (18)
C2—C3—C4—C5	−0.2 (3)	C12—C13—C14—N2	−0.7 (3)
C3—C4—C5—C6	0.7 (3)	O3—S1—C15—C21	−30.90 (19)
C4—C5—C6—C7	−0.5 (3)	O5—S1—C15—C21	89.57 (17)
C5—C6—C7—C2	−0.2 (3)	O4—S1—C15—C21	−150.81 (16)
C5—C6—C7—C8	−177.4 (2)	O3—S1—C15—C16	150.97 (16)
C3—C2—C7—C6	0.6 (3)	O5—S1—C15—C16	−88.55 (17)
C1—C2—C7—C6	−177.78 (19)	O4—S1—C15—C16	31.06 (19)
C3—C2—C7—C8	178.36 (18)	C21—C15—C16—C17	−0.7 (3)
C1—C2—C7—C8	−0.1 (2)	S1—C15—C16—C17	177.49 (15)
C1—N1—C8—O2	179.69 (19)	C15—C16—C17—C18	0.8 (3)
C9—N1—C8—O2	11.9 (3)	C16—C17—C18—C20	−0.1 (3)
C1—N1—C8—C7	−0.2 (2)	C16—C17—C18—C19	178.17 (19)
C9—N1—C8—C7	−168.02 (17)	C17—C18—C20—C21	−0.8 (3)
C6—C7—C8—O2	−2.3 (4)	C19—C18—C20—C21	−179.02 (19)
C2—C7—C8—O2	−179.7 (2)	C18—C20—C21—C15	1.0 (3)
C6—C7—C8—N1	177.6 (2)	C16—C15—C21—C20	−0.2 (3)
C2—C7—C8—N1	0.2 (2)	S1—C15—C21—C20	−178.34 (15)
C8—N1—C9—N2	−84.8 (2)		

### Hydrogen-bond geometry (Å, °)

**Table:**

*D*—H···*A*	*D*—H	H···*A*	*D*···*A*	*D*—H···*A*
C5—H5···Cg1^i^	0.95	2.70	3.531 (2)	147

Symmetry codes: (i) −*x*+1, −*y*, −*z*.
